# Association between marijuana use and kidney stone: a cross-sectional study of NHANES 2009 to 2018

**DOI:** 10.3389/fphar.2023.1214647

**Published:** 2023-09-07

**Authors:** Xingpeng Di, Liyuan Xiang, Menghua Wang, Xin Wei

**Affiliations:** ^1^ Laboratory of Reconstructive Urology, Department of Urology, Institute of Urology, West China Hospital, Sichuan University, Chengdu, Sichuan, China; ^2^ Department of Clinical Research Management, West China Hospital, Sichuan University, Chengdu, Sichuan, China

**Keywords:** kidney stone, marijuana, association, epidemiology, cross-sectional, National Health and Nutrition Examination Survey

## Abstract

**Objective:** The purpose of this investigation is to determine whether regular marijuana use is related to history of kidney stones in the US population.

**Methods:** Data were obtained from the National Health and Nutrition Examination Survey (NHANES) from 2009 to 2018. Kidney stone and marijuana use data were collected from self-report questionnaires. Multivariate logistic regression and multiple sensitivity analyses were applied to examine the relationship between marijuana usage and kidney stones.

**Results:** There are approximately 26.04% of the US population have admitted to using marijuana in their lifetime. Compared with none regular users, those with a higher frequency of marijuana use were more males, more non-Hispanic races, lower than high school education, overweight, no recreational activity, without diabetes mellitus, and more coronary heart disease. After adjusting for potential confounders, multivariate regression analysis demonstrated that marijuana use was inversely correlated to kidney stones in males (Odds ratio [OR] = 0.72, 95% Confidence interval [CI] = 0.54–0.97). One to seven times/week regular consumption of marijuana was associated with kidney stones in males (OR = 0.62, 95% CI = 0.43–0.89). Sensitivity analyses validated the robustness of our outcomes.

**Conclusion:** Our findings revealed that regular marijuana male users were inversely associated with kidney stones. Marijuana use one to six times/week was inversely related to the risk of kidney stones in males. Further studies are required to explore the dose and type associations of marijuana with kidney stones.

## Introduction

Marijuana use in the United States (US) has increased rapidly since 2005. As of 2017, marijuana use had been approved for medical use in 29 US states and Washington D.C. (Pacula and Smart, 2017). Currently, 18 US states approved legal recreational marijuana consumption. Although fewer researchers suggested marijuana use might have potential risk for human health, the impact of marijuana use on the human body was indeed controversial. Several studies focus on the relationship between marijuana use and kidney disease. For example, cannabis consumption was associated with an increased eGFR decline in patients with chronic kidney disease (Rein et al., 2023). However, the relationship is still controversial. An inverse association was also found between cannabis and urological cancer (Huang et al., 2023). However, rare studies focus on the impact of marijuana use and kidney stone.

Kidney stone is a life-long disease that causes heavy burden to patients (Thongprayoon et al., 2020). The incidence rate of kidney stones is 1.7%–14.8% (Romero et al., 2010). The prevalence of kidney stone disease increases by years with a recurrence rate of 50% (Siener and Hesse, 2021). The investigation between marijuana use and kidney disease were adjusted by demographic information such as age, gender, race/ethnicity, education level, and family income-to-poverty ratio, hypertension, and diabetes (Rein et al., 2023). Similarly, kidney stone is usually associated with age, gender, race/ethnicity, climate, occupation, metabolic diseases, and lifestyle (Torricelli et al., 2014). Furthermore, moderate physical activity has been reported to attenuate the association between ethylene oxide and kidney stone (Jiang et al., 2023). Hence, adjustment of these covariates is essential. Despite the wide investigation of kidney stone disease, the mechanisms in the formation of kidney stones is still understudied.

Given the wide consumption of marijuana in the US, many studies gradually focus on the function of marijuana in human health. However, it remains unknown whether marijuana has an impact on kidney stone disease. Therefore, we performed this study to explore the association between marijuana use and the risk of kidney stones using the National Health and Nutrition Survey (NHANES) dataset.

## Materials and methods

### Study population

This study included data from five cycles of NHANES from 2009 to 2018. The NHANES mainly includes interviews by well-trained interviewers to evaluate the health and nutritional status of individuals. NHANES enrolls sample data every 2 years utilizing a multistage, complex, stratified, and clustered probability design to better present the whole population of US (Centers for Disease Control and Prevention CDC, 2022). Adult participants (20–59 years old) with complete data on marijuana use and kidney stone history were included. Written informed consent was required from all participants. NCHS Ethics Review Board was approved the survey including humans (https://www.cdc.gov/nchs/nhanes/irba98.htm), and all the data were publicly available at https://www.cdc.gov/nchs/nhanes/.

### Assessment of marijuana use

The interview on the Drug Consumption questionnaire was used as the basis for evaluating marijuana use. The questions included: a) “Have you ever, even once, used marijuana or hashish?” and b) “Have you ever smoked marijuana or hashish at least once a month for more than 1 year?”. When the answers to both questions were “yes”, the individuals were thought to be frequent marijuana consumers. Non-users were those who responded “no” to the second question and had never smoked marijuana. In addition, “How often would you typically consume marijuana or hashish during the time that you smoked it?” was the question used to gauge marijuana use frequency. The frequency of marijuana use was classified as “less than one time per week”, “one to six times per week”, and “one or more times per day” in our study.

### Assessment of kidney stone history

The NHANES Kidney Conditions-Urology provides data on kidney stone history. The kidney stone history was assessed using the “Have you ever had kidney stones?”. Participants who answered “yes” to the question were considered patients with kidney stones. The participants with “no” answers were categorized as non-kidney stone patients.

### Covariates

#### Demographic confounders

Demographic characteristics were obtained from self-reported data, including age, gender, race/ethnicity, education level, and family income-to-poverty ratio. Based on previous studies (Chen et al., 2022; Di et al., 2023a), the race/ethnicity was classified as non-Hispanic Black, non-Hispanic White, Hispanic/Mexican, and other races. Education level mainly includes “less than 9th grade”, “9–11th grade”, “high school grade/GED or equivalent”, “Some college or AA degree”, “college graduate or above”, and others (Xie et al., 2022). The education level was divided by “high school grade/GED or equivalent”. The family income-to-poverty ratio (0–5) was categorized into tertiles (lowest T1 0–1.3; medium 1.3–3.5; and highest 3.5–5) (Ali et al., 2011).

#### Body mass index (BMI)

BMI data were calculated by weight/height^2^ (kg/m^2^) in the body measurement in NHANES. BMI was classified into <20 kg/m^2^, 20–25 kg/m^2^, 25–30 kg/m^2^, and over 30 kg/m^2^.

#### Smoking history

Smoking history was determined by “Have you smoked at least 100 cigarettes in your entire life?”. The participants who answered “yes” were considered smokers. Respondents who smoked less than 100 cigarettes were considered nonsmokers.

#### Recreational activity

The recreational activity evaluates the sports, fitness, and recreational activities intensity, which was classified as “none”, “moderate”, and “vigorous”. The standard was based on self-report question “In a typical week, do any moderate/vigorous sports, fitness, or recreational activities?”. If the answer to both questions was “Yes”, that was considered “vigorous recreational activity”. The data were obtained from the NHANES dataset by trained technicians.

#### Diabetes mellitus (DM)

DM was diagnosed by clinical guidelines: a) glycohemoglobin (HbA1c) > 6.5%; b) fasting glucose test greater ≥ 7.0 mmol/L; c) random blood glucose test ≥ 11.1 mmol/L; d) 2-h oral glucose tolerance test (OGTT) of blood glucose ≥ 11.1 mmol/L; e) diabetes medication or insulin history (Draznin et al., 2022).

#### Blood hypertension

Blood hypertension was classified as 140/90 mmHg.

#### Coronary heart disease

A diagnostic history of coronary heart disease was identified as a “yes” answer.

#### Menopausal status

Menopausal status data were obtained from self-reported question on reproductive health. Participants who answered “no” to the question “Have you had at least one menstrual period in the past 12 months?” were then asked, “What is the reason that you have not had a period in the past 12 months? (Pregnancy; breastfeeding; hysterectomy; menopause/change of life; other)” (Wang et al., 2021). Finally, 1095 postmenopausal females were included in analysis.

### Data analysis

We included strata, primary sampling unit, and sample weight in our analysis in accordance with the intricate, stratified design of the NHANES dataset to represent the whole population. Categorial variables were recorded by number count and percent, and continuous variables were expressed by mean ± standard deviation (SD).

A survey-designed logistic regression analysis was conducted to explore the association between marijuana use and kidney stone. Model 1 was adjusted for age, gender, race, education level, and family income-to-poverty ratio. Model 2 was adjusted for age, gender, race, education level, family income-to-poverty ratio, BMI, smoking history, recreational activity, DM, hypertension, and coronary heart disease. Model 3 was adjusted for adjusted for adjusted for age, gender, race, education level, family income-to-poverty ratio, BMI, smoking history, recreational activity, DM, hypertension, and coronary heart disease, which is used for detection of interaction in gender stratified analyses.

An additional 1669 observations were missing covariate data. Given systematic differences between participants with and without missing data, we performed multiple sensitivity analyses. The complete-case analysis excluded subjects with missing data in all covariates. Multiple imputation analysis (five imputations) indicated pooled estimate from five replications by the imputation of missing data (Su et al., 2011).

We used *R* software version 4.1 (http://www.R-project.org; The R Foundation) and EmpowerStats (http://www.empowerstats.com, X&Y Solutions, Inc., China) to analyze the data. A *p* < 0.05 (two-sided) was considered significant.

## Results

**FIGURE1:**
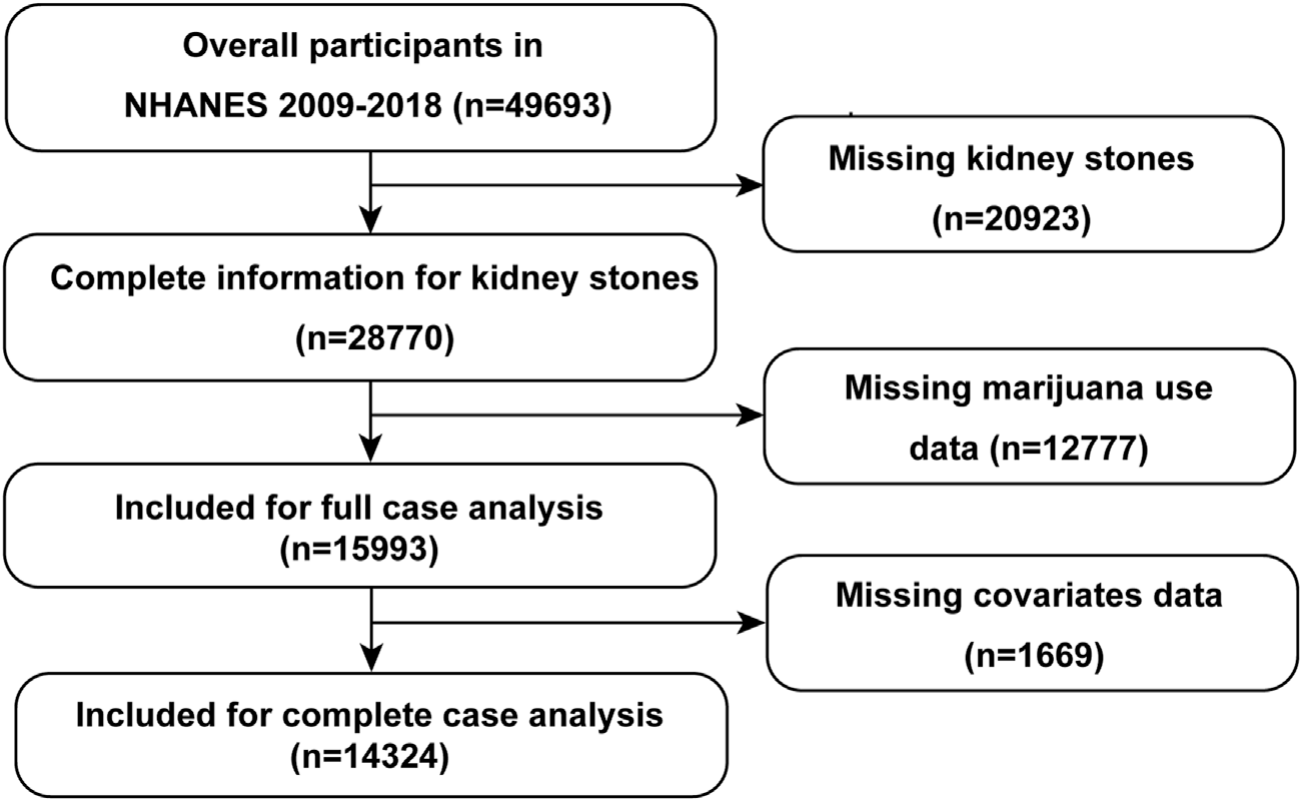
The flow chart of participant screening. NHANES, National Health and Nutrition Examination Survey.

Of 49693 participants included in our study, 20923 participants were excluded for missing kidney stone data, and 12777 participants were exlucded for missing data of marijuana use (Figure 1). Finally, 14324 subjects with full information were enrolled in our study. The detailed baseline characteristics missing data were recorded in Table 1. The age of participants was 39.26 ± 11.52 years old. The incidence rate of kidney stones was 7.83%. 4165/15993 (26.04%) of participants reported a marijuana use history in their lifetime. After stratified by kidney stone history, older, more non-Hispanic White, BMI ≥30 kg/m^2^, more smokers, no recreational activity, DM, hypertension, and more coronary heart disease were associated with kidney stone.

**TABLE1:** Baseline characteristics of 15993 participants aged 20–59 years from 2009–2018 NHANES.

Kidney stone	No	Yes	*p*-value
**Number**	14740	1253	
**Age**	38.92 ± 11.55	43.24 ± 10.39	<0.001
**Gender**			0.86
Male	7227 (49.03%)	605 (48.28%)	
Female	7513 (50.97%)	648 (51.72%)	
**Race**			<0.001
Non-Hispanic Black	3285 (22.29%)	170 (13.57%)	
Non-Hispanic White	5374 (36.46%)	632 (50.44%)	
Hispanic/Mexican	3841 (26.06%)	324 (25.86%)	
Other Races	2240 (15.20%)	127 (10.14%)	
**Education level**			0.45
≤ High school	5350 (36.30%)	472 (37.67%)	
> High school	9383 (63.66%)	781 (62.33%)	
Missing	7 (0.05%)	0 (0.00%)	
**Family income-to-poverty ratio**			0.53
<1.3	4473 (33.14%)	401 (34.30%)	
≥1.3, <3.5	4850 (35.93%)	416 (35.59%)	
≥3.5	4176 (30.94%)	352 (30.11%)	
**BMI (kg/m** ^**2**^ **)**			<0.001
<20	741 (5.03%)	44 (3.51%)	
≥20, <25	3764 (25.54%)	207 (16.52%)	
≥25, <30	4529 (30.73%)	369 (29.45%)	
≥30	5624 (38.15%)	621 (49.56%)	
Missing	82 (0.56%)	12 (0.96%)	
**Smoking history**			0.002
Non-smoker	8834 (59.93%)	663 (52.91%)	
Smoker	5902 (40.04%)	589 (47.01%)	
Missing	4 (0.03%)	1 (0.08%)	
**Recreational activity**			<0.001
None	6739 (45.72%)	687 (54.83%)	
Moderate	3561 (24.16%)	294 (23.46%)	
Vigorous	4440 (30.12%)	272 (21.71%)	
**Diabetes mellitus**			<0.001
No	12922 (87.67%)	997 (79.57%)	
Yes	1562 (10.60%)	248 (19.79%)	
Missing	256 (1.74%)	8 (0.64%)	
**Hypertension**			<0.001
No	10671 (72.39%)	722 (57.62%)	
Yes	4069 (27.61%)	531 (42.38%)	
**Coronary heart disease**			0.01
No	14576 (98.89%)	1222 (97.53%)	
Yes	144 (0.98%)	28 (2.23%)	
Missing	20 (0.14%)	3 (0.24%)	
**Marijuana Use**			0.40
No	10908 (74.00%)	920 (73.42%)	
Yes	3832 (26.00%)	333 (26.58%)	
**Marijuana Use Frequency**			0.81
<1 time/week	11684 (79.27%)	986 (78.69%)	
<1–7 times/week	1814 (12.31%)	146 (11.65%)	
≥7 times/week	1242 (8.43%)	121 (9.66%)	

Data were n (%) or mean ± SD; BMI, body mass index; NHANES, National Health and Nutrition Examination Survey.

### Weighted univariate and multivariate analyses

Since the consumption of marijuana was significantly higher in males than females, we directly performed gender-stratified logistic regression analyses. Collinearity tests were performed to confirm the non-collinear association among covariates. After adjusted for covariates, no significant difference was found between marijuana use and kidney stone (*p* > 0.05). After the model was adjusted for all confounders, interactive effect was found in gender stratified analysis (*p* = 0.046). Furthermore, gender-stratified logistic analysis showed a significant difference between marijuana use and kidney stone history in males (OR [Odds ratio] = 0.74, 95% Confidence interval [CI] = 0.56 to 0.97, *p* = 0.03) (Table 2). After adjusting for all covariates, marijuana use was inversely correlated to kidney stones in males (OR = 0.72, 95% CI = 0.54 to 0.97, *p* = 0.03). No significant differences were found in overall and female participants.

**TABLE 2:** Weighted univariate and multivariate logistic regression analyses between marijuana use and history of kidney stone(s), gender stratification.

		Kidney stone		
Gender	Marijuana use	Model 1	Model 2	Model3
		OR (95% CI), *P*	OR (95% CI), *P*	OR (95% CI), *P*
Overall	No	Reference	Reference	Reference
	Yes	0.91 (0.75,1.14), 0.33	0.89 (0.73,1.11), 0.32	0.74 (0.55,0.99), 0.046
Male	No	Reference	Reference	
	Yes	0.74 (0.56,0.97), 0.03	0.72 (0.54,0.97), 0.03	—
Female	No	Reference	Reference	—
	Yes	1.16 (0.90,1.49), 0.25	1.14 (0.87,1.50), 0.33	—

Model1: adjusted for age, gender, race, education level, and family income-to-poverty ratio. Model2: adjusted for age, gender, race, education level, family income-to-poverty ratio, BMI, smoking history, recreational activity; DM, hypertension, and coronary heart disease. Model3: adjusted for adjusted for age, gender, race, education level, family income-to-poverty ratio, BMI, smoking history, recreational activity; DM, hypertension, and coronary heart disease, which is interacted with gender. For the models in which the analysis was stratified by gender, gender was not included as a covariate. *p* < 0.05 presents significant difference. BMI, body mass index; CI, confidence interval; DM, diabetes mellitus; OR, odds ratio.

In a further analysis stratified by frequency of marijuana use, interactive effect was found in gender stratified analysis in one to seven times a week consumption of marijuana group (*p* = 0.02) (Table 3). In gender stratified analysis, one to seven times a week consumption of marijuana was associated with a lower risk of kidney stones in males (Model 1, OR = 0.64, 95% CI = 0.46 to 0.90, *p* = 0.01) (Table 3). Model 2 (OR = 0.62, 95% CI = 0.43 to 0.89, *p* = 0.01) indicated similar results. Furthermore, we analyzed the association between the frequency of marijuana use and kidney stone in postmenopausal females to investigate whether hormone level plays a role in the relationship between marijuana use and kidney stone in females. The average age of postmenopausal females was 52.26 years old. However, the results showed no significant difference still (Supplementary Table S1).

**TABLE 3:** Weighted univariate and multivariate logistic regression analyses between frequency of marijuana use and history of kidney stone(s), gender stratification.

		Kidney stone		
Gender	Marijuana use frequency	Model 1	Model 2	Model 3
		OR (95% CI), *P*	OR (95% CI), *P*	OR (95% CI), *P*
Overall	<1 time/week	Reference	Reference	Reference
	1–7 times/week	0.89 (0.68,1.16), 0.37	0.88 (0.66,1.17), 0.37	0.64 (0.45,0.91), 0.02
	≥7 times/week	1.07 (0.80,1.42), 0.65	1.05 (0.79,1.38), 0.74	0.89 (0.60,1.31), 0.53
Male	<1 time/week	Reference	Reference	Reference
	1–7 times/week	0.64 (0.46,0.90), 0.01	0.62 (0.43,0.89), 0.01	—
	≥7 times/week	0.91 (0.62,1.34), 0.63	0.89 (0.60,1.32), 0.56	—
Female	<1 time/week	Reference	Reference	Reference
	1–7 times/week	1.27 (0.88,1.84), 0.19	1.29 (0.86,1.92), 0.21	—
	≥7 times/week	1.33 (0.95,1.87), 0.19	1.31 (0.93,1.84), 0.12	—

Model1: adjusted for age, gender, race, education level, and family income ratio. Model2: adjusted for age, gender, race, education level, family income-to-poverty ratio, BMI, smoking history, recreational activity; DM, hypertension, and coronary heart disease. Model3: adjusted for adjusted for age, gender, race, education level, family income-to-poverty ratio, BMI, smoking history, recreational activity; DM, hypertension, and coronary heart disease, which is interacted with gender. For the models in which the analysis was stratified by gender, gender was not included as a covariate. *p* < 0.05 presents significant difference. BMI, body mass index; CI, confidence interval; DM, diabetes mellitus; OR, odds ratio.

### Sensitivity analyses

To validate our outcomes, we conducted sensitivity analyses. Marijuana use was inversely associated with kidney stones in males in complete-case analysis (OR = 0.72, 95% CI = 0.54 to 0.97, *p* = 0.03) and multiple imputation analysis (OR = 0.71, 95% CI = 0.53 to 0.95, *p* = 0.02) (Supplementary Table S2). Marijuana use between one and seven times a week was also associated with a low risk of kidney stones in males (Supplementary Table S3). The OR for complete-case analysis was 0.62 (95% CI = 0.43 to 0.89, *p* = 0.01). The multiple imputation analysis showed a similar outcome (*p* = 0.01).

## Discussion

Based on this nationally well-established design of the US population, we explored the association between marijuana use and kidney stone. Consistent with previous studies (Zhu et al., 2022), 26.04% of adult participants reported a marijuana use history. Our findings suggested that marijuana use was inversely associated with kidney stones in males. Furthermore, we found a regular marijuana use (<6 times/week) indicated a negative relationship with kidney stones in the male population. However, no such differences were found in the overall and female populations.

Since the consumption of marijuana is not accessible in most countries, limited data investigate the relationship between marijuana use and diseases. Our negative findings about the impact of marijuana use on kidney stones could be explained with theories of the stone formation process. Previous studies demonstrated that cannabinoid application increased urine output without affecting the excretion of Cl^−^ or K^+^ in mice (Chopda et al., 2013). The diuretic effects of cannabinoids shorten the time of crystal remaining in the kidney, thereby decreasing the risk of kidney stone formation. Furthermore, cannabidiol, a main component of cannabis, exerts benefits in anti-inflammatory and antioxidant effects (Atalay et al., 2019). Randall’s plaque presented on kidney papillary surfaces is identified to attach Calcium oxalate. The pro-inflammatory processes were found surrounding Randall’s plaques (Khan et al., 2021). In addition, crystal deposition in the kidney is also related to the generation of reactive oxygen species (ROS), and inflammasome activation. Hence, it is speculated that cannabidiol has natural advantages in attenuating inflammatory responses and reducing oxidative stress.

Several studies demonstrated that early use of marijuana was inversely associated with the risk of many diseases, such as cognition impairment, psychosocial symptoms, depression, cardiovascular health, and others (Jivanji et al., 2020). However, marijuana use has been reported a positive relationship with diabetes mellitus (Sidney, 2016). Unfortunately, marijuana users also suffer from a high risk of depression, anxiety, and others. In addition, the efficacy of marijuana in alleviating pain was identified as an approach to treating refractory pain in the human body. Due to the allowance of recreational marijuana consumption in the US, researchers focused more on the potential risk of marijuana use for multiple diseases. Studies suggested that marijuana use contributed to memory impairment, social skills and judgment impairment, cognitive injury, chronic bronchitis, and psychosis disorders (Volkow et al., 2014). Some retrospective studies revealed that cannabis might inversely influence the cardiovascular system (Ghosh and Naderi, 2019). However, a systematic review of 24 studies investigated the associations between marijuana use and cardiovascular risk factors (Ravi et al., 2018). Despite the metabolic benefits of marijuana use in cross-sectional studies, no such findings were shown in prospective studies. Interestingly, the relationship between marijuana use and cardiovascular diseases was insufficient. In addition, several laboratory models show the anti-inflammatory potential of cannabis but lack evidence in humans. However, although cannabis research is at an early stage, the therapeutic potential of cannabis should not be ignored (Naftali, 2020). In addition, marijuana has also been reported to alleviate some refractory neurogenic symptoms by reducing urge incontinence, and nocturia and improving urinary bladder control (Kavia et al., 2010), which may influence the mechanotransduction of bladder (Di et al., 2023b). Another study indicated that marijuana users had a low risk of lower urinary tract symptoms compared to nonusers (Fantus et al., 2019). Intriguingly, we found no association between more frequent marijuana use (≥7 times/week) and kidney stones, whereas a relationship exists between less frequent marijuana use (1–7 times/week) and kidney stones in males. A study revealed that less to moderate alcohol intake is associated with lower risk of cardiovascular events (Biddinger et al., 2022). However, after adjusting lifestyle, this association is weakened. Therefore, the impact of marijuana on the human body is still controversial and further studies are warranted.

Unfortunately, our study found no association between marijuana use and kidney stone in females. Despite further analyses in postmenopausal females, marijuana use was still unassociated with kidney stone in females. Hence, we hypothesized that hormone level could not regulate this association. A systematic review has demonstrated that menstrual cycle appeared to be a specific factor influencing some addictive behaviors (Joyce et al., 2021). However, the association between menstrual cycle and kidney stone has not been fully clarified. In addition, a previous study demonstrated that lower urinary saturation of stone-forming crystals might be the reason for less kidney stone formation in females (Heller et al., 2002). The explanation suggested that estrogen might be associated with a lower risk of kidney stones. Inversely, postmenopausal females had a higher risk of kidney stones. It is speculated that estrogen might overwhelm the impact of marijuana use on kidney stones. Therefore, marijuana use might have an insufficient association with the risk of kidney stones in the female population.

To our knowledge, this study is the first cross-sectional study to explore the association between marijuana use and the risk of kidney stones from the population-based NHANES dataset. We comprehensively included marijuana use history and frequency of marijuana use in the US population, and we enrolled in the most crucial confounders in our logistic analyses. We further confirmed our findings with several sensitivity analyses, including complete-case and multiple imputation analyses.

It should be noted that there are also some limitations in our study. Due to the cross-sectional design of the NHANES dataset without medical limitation, the causal link between marijuana use and kidney stone cannot be obtained. Notably, we introduced family income-to-poverty ratio for adjustment. However, race and ethnicity are associated with socioeconomic status that is related to multiple health outcomes (Kaufman and Cooper, 2001). Socioeconomic status may be a causal intermediate between race and kidney stones. Hence, the analyses have the limitation for overadjustment bias (Schisterman et al., 2009). Moreover, the missing data of participants may cause selection bias in analyses that may affect the interpretation of the results. Although we performed a sensitivity analyses using data including missing data, more detailed analyses with full cases is more advisable in complex database processing, as well as domain analysis (Seidenberg et al., 2023). The marijuana use information was collected by self-reported approach without the exact type and dose of cannabis as well as a specific biomarker in exposure assessment, which might also cause bias. In addition, lacking a dose of marijuana use, we are not able to assess the total dose of marijuana use, which is especially important in this study. Therefore, future studies should concentrate more on the dose and types of marijuana use. More prospective and large-scale studies are needed to further investigate the association between marijuana or its components use and kidney stone.

## Conclusion

Taken together, our findings suggested that regular marijuana male users were related to a lower risk of kidney stones. Marijuana use for one to six times/week was inversely associated with the risk of kidney stones in males. Further studies are warranted to investigate the dose and type associations of marijuana with kidney stones.

## Data Availability

Publicly available datasets were analyzed in this study. This data can be found here: https://www.cdc.gov/nchs/nhanes/.
