# Supplementary material for: Association between marijuana use and kidney stone: a cross-sectional study of NHANES 2009 to 2018
Source: Front Pharmacol. 2023 Sep 7;14:1214647. doi: 10.3389/fphar.2023.1214647 (PMC10513173; doi:10.3389/fphar.2023.1214647)
Supplement: Supplementary file 1 [file DataSheet1.docx]

**Supplementary Materials**

**Table S1** Weighted logistic regression analyses between frequency of marijuana use and kidney stone in postmenopausal females.

| Marijuana Use | Model 2 OR (95% CI), P |
| --- | --- |
| < 1 time/week | Reference |
| 1-7 times/week | 0.922 (0.501,1.696), 0.790 |
| ≥ 7 times/week | 1.177 (0.612,2.267), 0.620 |

Adjusted for age, gender, race, education level, family income ratio, BMI, smoking history, recreational activity, DM, hypertension, and coronary heart disease. *P* < 0.05 presents significant difference. BMI, Body mass index; CI, Confidence interval; DM, Diabetes mellitus; OR, Odds ratio.

**Table S2.** Sensitivity analyses among complete case, full case, and multiple imputation of marijuana use.

|  |  | Kidney stone | | |
| --- | --- | --- | --- | --- |
| Gender | Marijuana Use | Complete case OR (95% CI), *P* | Full case OR (95% CI), *P* | Multiple imputation OR (95% CI), *P* |
| Overall | No | Reference | Reference | Reference |
|  | Yes | 0.899 (0.726,1.113), 0.321 | 0.866 (0.703,1.066), 0.171 | 0.874 (0.711,1.075), 0.198 |
| Male | No | Reference | Reference | Reference |
|  | Yes | 0.723 (0.537,0.973), 0.033 | 0.716 (0.537,0.953), 0.026 | 0.710 (0.529,0.953), 0.023 |
| Female | No | Reference | Reference | Reference |
|  | Yes | 1.143 (0.871,1.500), 0.330 | 1.086 (0.836,1.410), 0.532 | 1.110 (0.856,1.441), 0.425 |

Adjusted for age, gender, race, education level, family income ratio, BMI, smoking history, recreational activity, DM, hypertension, and coronary heart disease. *P* < 0.05 presents significant difference. BMI, Body mass index; CI, Confidence interval; DM, Diabetes mellitus; OR, Odds ratio.

**Table S3.** Sensitivity analyses among complete case, full case, and multiple imputation of frequency of marijuana use.

|  |  | Kidney stone | | |
| --- | --- | --- | --- | --- |
| Gender | Marijuana Use | Complete case OR (95% CI), *P* | Full case OR (95% CI), *P* | Multiple imputation OR (95% CI), *P* |
| Overall | < 1 time/week | Reference | Reference | Reference |
|  | 1-7 times/week | 0.879 (0.660,1.172), 0.374 | 0.855 (0.644,1.136), 0.274 | 0.864 (0.653,1.145), 0.303 |
|  | ≥ 7 times/week | 1.047 (0.794,1.382), 0.740 | 0.986 (0.754,1.288), 0.915 | 1.002 (0.770,1.304), 0.988 |
| Male | < 1 time/week | Reference | Reference | Reference |
|  | 1-7 times/week | 0.619 (0.433,0.886), 0.010 | 0.629 (0.444,0.892), 0.012 | 0.626 (0.440,0.891), 0.010 |
|  | ≥ 7 times/week | 0.889 (0.597,1.323), 0.556 | 0.829 (0.564,1.216), 0.341 | 0.831 (0.563,1.225), 0.343 |
| Female | < 1 time/week | Reference | Reference | Reference |
|  | 1-7 times/week | 1.273 (0.881,1.839), 0.194 | 1.219 (0.826,1.797), 0.323 | 1.246 (0.841,1.847), 0.268 |
|  | ≥ 7 times/week | 1.285 (0.862,1.916), 0.214 | 1.265 (0.910,1.759), 0.168 | 1.303 (0.930,1.825), 0.121 |

Adjusted for age, gender, race, education level, family income ratio, BMI, smoking history, recreational activity, DM, hypertension, and coronary heart disease. *P* < 0.05 presents significant difference. BMI, Body mass index; CI, Confidence interval; DM, Diabetes mellitus; OR, Odds ratio.
